# Female homicides and femicides in Ecuador: a nationwide ecological analysis from 2001 to 2017

**DOI:** 10.1186/s12905-022-01839-2

**Published:** 2022-06-27

**Authors:** Esteban Ortiz-Prado, Paola Villagran, Ana Lucia Martinez-Abarca, Aquiles R. Henriquez-Trujillo, Katherine Simbaña-Rivera, Lenin Ana M. Gómez-BarrenoDiaz, Carla E. Moyano, Vanessa Arcos-Valle, Maria Dolores Miño, Sara A. Morgan

**Affiliations:** 1 One Health Research Group, Faculty of Health Science, Calle de los Colimes Y Avenida De los Granados, Universidad de Las Americas, Quito, 170137 Ecuador; 2 Director of the Observatory of Human Rights and Justice, Quito, Ecuador; 3 School of Law, Universidad Internacional del Ecuador, Quito, Ecuador; 4 School of Primary Care, Population Sciences and Medical Education, Faculty of Medicine, Southampton General Hospital, , University of Southampton, Tremona Road, Southampton, SO16 6YD UK

**Keywords:** Female homicide, Femicide, Crime, Gender-based violence, Violence, Gender

## Abstract

**Background:**

Gender–based violence is a major public health concern arising from the structural discrimination of women and girls. In 2014, Ecuador criminalized acts of femicide in response to a growing crisis across the region. As no epidemiological studies on the state of female homicides and femicides have been published, we estimated patterns of female homicides and femicides nationally and the burden through economic cost per years of life lost, between 2001 and 2017.

**Methods:**

Using aggregated data from the National Institute of Census and Statistics and police records we estimated the annual mortality rates, cumulative incidence and prevalence odds ratios for female homicides and femicides, from 2001 to 2017. The impact of aggressions, assaults and violence on years of life lost due to premature mortality was estimated using the Human Capital method.

**Results:**

Over the period, at least 3236 cases of female homicides and femicides were reported. The highest murder rate occurred in the province of Sucumbíos (6.5 per 100,000) and in the Putumayo canton (12.5 per 100,000). The most common way to murder their victims was using firearms (38%). The highest odds ratio was estimated for women aged between 25 and 29, at 4.5 (3.9–5.1), of primary school attainment at 17.2 (14.6–20.3) and of Afro-Ecuadoran descent 18.1 (10.5–30.9). Female homicide-related costs reached, on average, $35 million per year and more than $500 million lost from 2001 to 2017.

**Conclusions:**

The high rates, distribution and cost indicate that investments are urgently needed to address the structural causes and reduce the impact of female homicides and femicides in Ecuador; thereby protecting the livelihood and well-being of their women and girls.

**Supplementary Information:**

The online version contains supplementary material available at 10.1186/s12905-022-01839-2.

## Introduction

Gender–based violence is a major public health concern and violation of women’s human rights that affects communities all over the world [1]. An estimated 1.2 billion women–30% of the female population–have experienced either physical and/or sexual intimate partner violence or non–partner sexual violence in their lifetime [2]. When such violence results in an assassination, or femicide*,* societies experience the most extreme form of violence against women [3]. Several human rights bodies have characterized femicides as a particular type of crime, that occurs in the ‘context of structural discrimination against women and girls’ [4]. Femicides are therefore not isolated occurrences; they result from systematic and structural phenomenon, enrooted in social behaviours and accepted stereotypes [5, 6]. Studies attribute several risk and protective factors to the risk of a femicide occurring. These include intra–familial relationships, intimate partner violence, alcohol or drugs consumption, level of education, poverty, religious beliefs and other cultural aspects, that vary from region to region [5, 7–12].

Global estimates suggest that at least 87,000 women were assassinated in 2017, representing more than six women killed every hour [3]. The African continent has the highest standardised murder rate with 1.6 per 100,000 cases, followed by the Americas with 1.3 per 100,000 and Oceania with 1.3 per 100,000 [3]. Empirical evidence on the global demographics and epidemiological distribution still remain rare [2]. In Latin America, a region with high levels of civil violence, very few countries have reported the overall impact of violence towards women at a national level [13–16]. Two previous studies used data from a national network of non-governmental organisations, the network of shelters for women victims of violence and the network of external care centres for women victims of violence, to produce estimates in Ecuador. Over 2 years (2018–2019) the prevalence of femicide in the Ecuadorian female population aged 15 + was estimated at 0.8 per 100,000 [17], a decrease from a study on the previous year (2017) reporting a rate of 2.4 per 100,000 across the provincial level [18]. In 2014, Ecuador criminalised femicide through the inclusion of Article 141 of the Organic Criminal Code (OCC). As one of the last countries in the region to provide such protection to women and girls, this was an important milestone and the result of intense lobbying and activism by women´s rights advocates and organizations. The legislation for femicide has become intimately linked with acts of gender–based violence in Ecuador and [19] states that “the person who, as a result of power relations manifested in any type of violence, kills a woman due to the fact that she is or because of her gender, they will be punished with imprisonment from 22 to 26 years*”* [20].

In Ecuador, although sociological manuscripts, legal interpretations and other historical evidence are available, a long-term epidemiological study of the prevalence and economic burden of femicide has never been attempted. This study aims to address the gap in the literature on crimes against women, specifically female homicides and femicides, occurring in Ecuador from 2001 to 2017; thereby creating further awareness on a stark reality that, until now, has been poorly analysed within the national context. Specifically, we aim to examine the social, economic, geographical and demographic patterns of female homicides and femicides nationally and the burden of these through economic cost per years of life lost.

## Methods

### Data source

We extracted aggregated mortality data on female homicides and male homicides from the National Institute of Census and Statistics (INEC) of Ecuador, which is responsible for generating and reporting the official national statistics for decision making in public policy. The country is divided into four geographical regions: the coastal region; the highlands or sierra region; the amazon region and the insular region (Galapagos Islands). The extracted data included available information (2001–2017) from the 24 provinces and the 223 cantons in the country, where cantons are political subdivision of a province in the country. In 2017 the population of Ecuador was estimated at 16,624,424 inhabitants based on the latest available census data from 2010 and its projections [21]. The dataset contains information on age, civil status, educational status, ethnicity, date of death, gender, place of registration (urban or rural) and the underlying cause of death coded using the International Classification of Diseases, tenth revision (ICD-10). In Ecuador, all violent deaths, are subject to an obligatory autopsy, an expert investigation and a medical certificate of death. With this information, the ICD-10 code is assigned to each death. However, since 2014, when the law changed, the term femicide was also included as a cause of death within the death certificates, but not in the ICD-10 code. To validate the data, comparisons were made against the official records held by the national police to ensure accuracy in reporting through the INEC database. The dataset is readily available as comma-separated values (CSV) or dBase database file (DBF) format in the public INEC’s domain: https://aplicaciones3.ecuadorencifras.gob.ec/sbi-war/

### Data analysis

Using the ICD– 10 classifications (see Additional file 1: Appendix), we estimated the annual mortality rate for female homicides and femicides per 100,000 population. These were age-standardized using projection data, by canton and province, according to the available information from the INEC Census in 2010 [19]. The term ‘female homicides and femicides’ have been used as a catch–all term acknowledging that prior to 2014 when the legislation was introduced, a proportion of female homicides were, by definition, also femicides. Following 2014, as the ICD-10 did not include the term femicides, we were unable to make accurate estimations on femicide alone. We made comparisons with the official databases held by the police and obtained the proportion of homicides that were also considered as femicides, reporting the prevalence of femicides from 2014 onwards. Mortality rates were calculated by dividing the number of new cases per year, by the total population at risk during each year. We calculated the rates for female homicides and femicides per 100,000 population and 95% CI by 5–year age group (5–9, 10–14, 15–19, 20–24, 25–29… 65 years and older), compared to the youngest age group (0– 4 years), and to male homicides within the same period. Analysis of odds ratio of female homicides and femicides, by social, economic and demographic variables, were calculated using ethnicity (indigenous, afro-ecuadorian/ afro-descendant, montubia, mixed, white, other), highest educational attainment achieved (primary school, secondary school (incomplete), high school, postgraduate), and civil status (united by law, single, married, divorced, separated, widowed). Trends by region and province were estimated using cumulative incidence across the years 2001–2017. Cumulative incidence was estimated by dividing the number of cases with the population at risk (gender and age per year) and then producing a final average estimate.

The ICD– 10 codes for assault and aggression (see Appendix) were categorized into nine groups representing underlying cause of death by: chemicals, strangulation, drowning, sexual aggressions, firearms, fire-related mechanisms, sharp objects (e.g. knife), blunt objects and other. Comparison of cause of female deaths against cause of male deaths were made using these codes. All statistical analysis accepted significance with a p-value < 0.05. Calculations were completed using IBM SPSS Statistics for Windows, Version 24·0 and R Core Team software 2018 version 3.5.1. Cartography was generated using QGIS Development Team, version 2.8.

### Burden of female homicides and femicides

To analyse the impact of female homicide and femicides we calculated the overall impact of aggressions, assaults and violence on years of life lost due to premature mortality (YLL). YLL were calculated as the number of deaths multiplied by life expectancy of the woman at the age of death, considering a life expectancy at birth of 86.2 years according to the Global Burden of Disease. This methodology has been described by Murray and colleagues and previously used in similar analysis; no time discount, nor age weighting were added to the calculations [22]. Indirect costs were calculated using the human-capital (HC) method to value productivity losses. The HC method considers the person’s hours of productivity that are lost until their retirement age. Since it was not possible to ascertain the level of income of each of the deceased women, we based our estimates at the official basic salary for women of productive age at the time of their death. The official minimum wage, which varies accordingly to the GDP per capita, ranges from $370 to $390 USD per month. To estimate the total productivity losses, we multiplied the number of YLL in woman aged 15 to 64 years old by the official GDP per capita at the age of death.

## Results

**Fig. 1:**
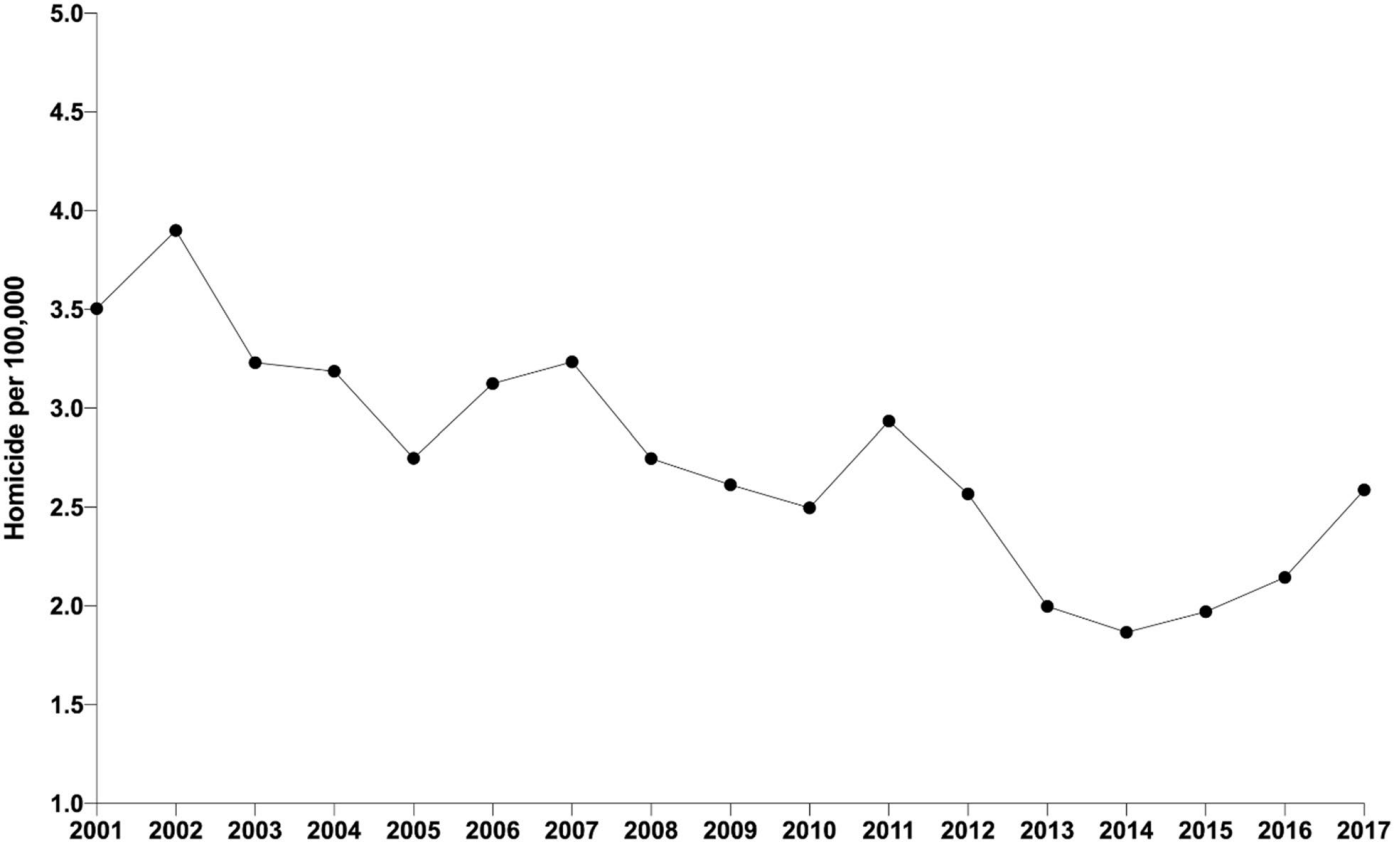
Annual female homicide and femicide rate per 100,000 population in Ecuador, 2001–2017. Footnote: This Figure is the incidence rate of female homicide and femicide from 2001 to 2017; the total number divided by the female population in each year

We analysed data from 17 years of available data (2001–2017), which included 3236 cases of female homicides and femicides. Since 2014, where femicide began to be classified as such, 249 cases of femicide have been registered, increasing from 18% in 2014 to 57% in 2017. The annual homicide rate per 100,000 varied from 1.8/100,000 to 3.8/100,000 (Fig. 1).

The majority of deaths occurred among young women between 20 and 30 years old, representing more than 28% of the total number of violent deaths amongst women. The mean age of women in the overall period was 33. The youngest victims were observed in the group of sexual related assaults (10 years median 95% CI, 6–16) and the oldest victims were killed by blunt objects (33 years median 95% CI, 28–44). The youngest victim reported was 1– year– old and the oldest 99 years.

**Fig. 2:**
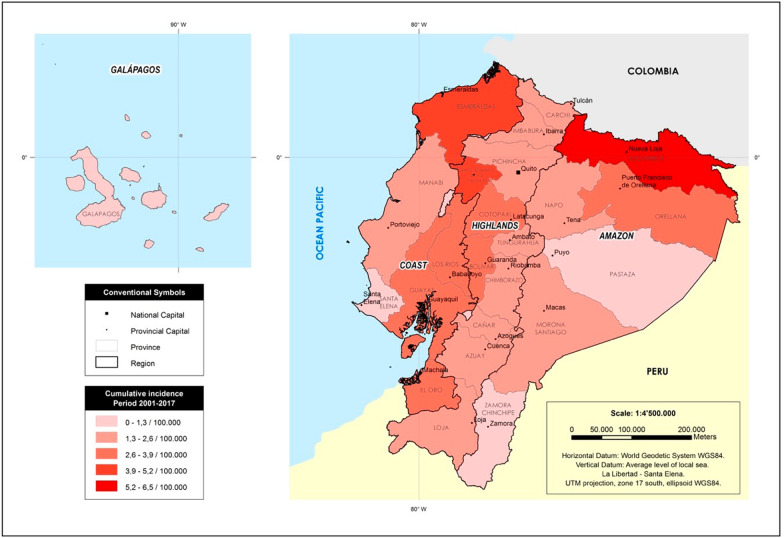
Cumulative incidence of female homicides and femicides by region and province, 2001–2017. Footnote: This Figure is a map of the country with the calculated incidence rate per 100,000 inhabitants by province of residence

Female homicides and femicides were most prevalent in urban areas (84%–*N* = 2718) compared to rural areas. The region with the highest cumulative incidence rate per 100,000 people was the amazon region (48.1/100,000), followed by the coast (43.2/100,000), the highlands (31.3/100,000) and the Galapagos islands, where no murders against women have been reported. Of the 24 provinces, the highest rate was Sucumbíos (6.5/100,000), Esmeraldas (4.6/100,000), Santo Domingo de los Tsachilas (4.3/100,000), Bolivar (3.8/100,000), and El Oro (3.6/100,000) (Fig. 2).

**Fig. 3:**
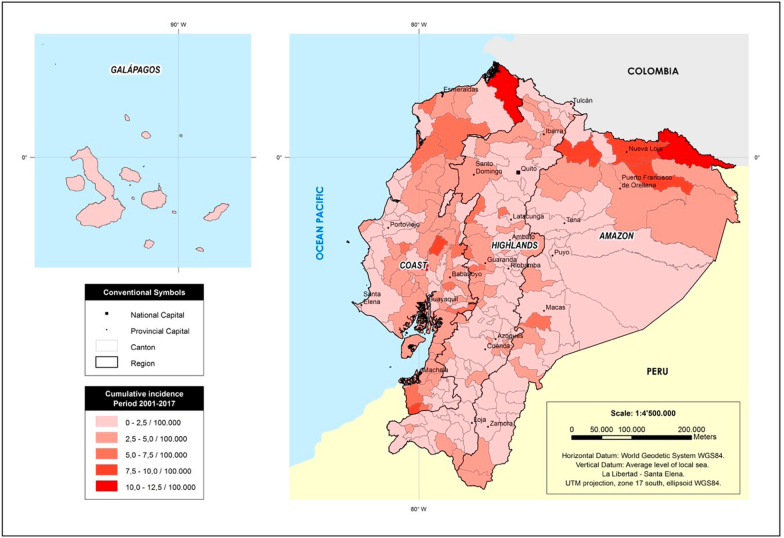
Cumulative incidence of female homicides and femicides by region and canton, 2001–2017. Footnote: This Figure is a map of the region and the canton level (smaller than provincial level) with calculated incidence rate per 100,000 inhabitants by province of residence

Of the 223 cantons, the top five most violent in terms of the overall (2001–2017) the female homicide and femicide rate per 100,000 were Putumayo (12.5/100,000), San Lorenzo (11.1/100,000), Palestina (10.5 /100,000), Las Lajas (9.9/100,000) and Lago Agrio (9.3/100,000) (Fig. 3). In the overall 17 years of data, 32 cantons did not report any homicide or femicide.

Compared to the youngest group, women from 25 to 29 years old had the highest rate at 7.0 (95% CI 5.6–8.8). (Table 1) Compared to males, females had a lower odds ratio of homicide at each age category, although this difference was less apparent amongst younger age groups.

**Table 1:** Rate of female homicide and femicide in Ecuador by age category, 2001–2017

Age group	Population	Cases (n)	Rate per 100,000 population [CI 95%]	|Odds ratio compared to lowest age group [CI 95%]	Odds ratio compared to male homicides at same age group [CI 95%]
0–4	826,528	89	0.68 [0.48–0.88]	Ref	0.79 [0.58–1.09]
5–9	799,393	92	0.73 [0.47–1.00]	1.07 [0.79–1.43]	0.84 [0.61–1.14]
10–14	759,419	106	0.90 [0.60–1.15]	1.30 [0.97–1.71]	0.54 [0.41–0.71]*
15–19	708,354	331	2.56 [2.08–3.03]	4.34 [3.43–5.48]*	0.14 [0.12–0.16]*
20–24	656,390	450	4.14 [3.68–4.61]	6.36 [5.06–7.98]*	0.09 [0.08– 0.11]*
25–29	612,107	463	4.47 [3.86–5.08]	7.02 [5.59–8.80]*	0.09 [0.08–0.11]*
30–34	562,150	342	3.50 [2.93–4.07]	5.65 [4.47–7.13]*	0.07 [0.06–0.08]*
35–39	501,112	290	3.47 [2.91–4.02]	5.37 [4.23–6.81]*	0.09 [0.08– 0.12]*
40–44	442,537	260	3.48 [2.93–4.04]	5.45 [4.28–6.93]*	0.12 [0.08–0.11]*
45–49	386,889	199	3.28 [2.60–3.97]	4.78 [3.71–6.13]*	0.90 [0.08–0.11]*
50–54	326,634	149	2.70 [2.10–3.29]	4.23 [3.25–5.50]*	0.11 [0.08–0.12]*
55–59	266,079	93	1.92 [1.44–2.40]	3.24 [2.42–4.33]*	0.09 [0.07–0.12]*
60–64	212,730	85	2.34 [1.69–2.98]	3.71 [2.75–4.99]*	0.13 [0.11–0.17]*
> 65	508,031	286	2.57 [2.09–3.05]	4.10 [3.01–5.58]*	0.16 [0.12–0.21]*
Total	7,568,353	3235	N/A	N/A	N/A

^*^p-value *** < 0.05; ** < 0.01; * < 0.001

Compared to those who completed postgraduate education, the odds ratio of female homicides and femicides was 17.2 (95% CI 14.6–20.3) for those that attended primary school. (Table 2) Being of Afro-Ecuadorian/ Afro- descent had an odds ratio of 18.1 (95% CI 10.5–30.9) compared to being of White ethnicity.

**Table 2:** Prevalence and prevalence odds ratio of female homicide and femicide in Ecuador with social, economic and demographic variables

	Cases, n (%)	Odds ratio compared to reference category [CI 95%]
*Civil status*		
United by law ^i^	573 (18)	0.66 [0.60–0.73]
Single	1419 (44.0)	Ref
Married	850 (26.0)	0.62 [0.56–0.67]
Divorced	105 (3.0)	1.05 [0.86–1.28]
Separated	27 (1.0)	0.10 [0.07–0.15]
Widowed	263 (8.0)	1.08 [0.95–1.23]
*Highest level of educational attainment*		
None	276 (8.5)	0.82 [0.67–0.99]
Primary school	1102 (34.1)	17.2 [14.6–20.3]
Incomplete secondary school	866 (26.7)	0.25 [0.21–0.30]
Complete High school	105 (3.3)	0.08 [0.07–0.11]
Postgraduate	161 (5.0)	Ref
No information	726 (22.4)	4.49 [3.78–5.33]
*Ethnicity*		
Indigenous	75 (2.3)	4·64 [2.62–8.21]
Afro-Ecuadorian / Afro-descendant	290 (8.9)	18·1 [10.5- 30.9]
Montubia	9 (0.3)	0·58 [0.24–1.33]
Mixed	979 (30.3)	5·91 [3.49–10.0]
White	14 (0.4)	Ref
Other	4 (0.1)	5·25 [1.72–15.96]
No information	1865 (57.6)	N/A

^i^ Ecuador being married entitles all the civil rights, whereas united by law is a legal status acquired after the couple have spent two years living together and on signing a free union form

The most common cause of death were firearms (38%), followed by sharps objects (30%) and strangulation (14%) (Table 3). Compared to males, females had a 4·33 odds ratio (95% CI 1.63–11.4) of death due to sexual aggressions.

**Table 3:** Cause of death

	Cases (n)	Age mean [CI 95%]	Age median [CI 95%]	Odds ratio compared to males [CI 95%]
Chemicals	46 (1)	25 [19–32]	22 [19–32]	0.10[0.06–0.17]
Strangulation	444 (14)	36 [34–39]	28 [26–32]	0.42[0.37–0.46]
Drowning	16 (0)	38 [29–46]	26 [22–42]	0.06[0.03–0.10]
Sexual Aggressions	22 (1)	25 [9–40]	10 [6–16]	4.33[1.63–11.4]
Firearms	1222 (38)	33 [32–34]	30 [29–32]	0.06[0.06–0.06]
Fire related	18 (1)	34 [19–49]	30 [17–45]	0.36[0.21–0.62]
Sharp Objects	960 (30)	36 [35–37]	32 [31–34]	0.15[0.14–0.16]
Blunt Object	83 (3)	39 [33–45]	33 [28–44]	0.17[0.13–0.21]
Others	425 (13)	37 [34–39]	31 [29–34]	0.27[0.24–0.29]
Total	3263	33.6	26.8	N/A

In most cases of female homicides and femicide in Ecuador, women were young. The vast majority of women killed were under 35 years of age (57·9%), so the economic burden due to lost lives is significant, reaching an average of 9,675 years of life lost prematurely per year, and more than 164,000 years in the last 17 years of available data. Female homicide and femicide–related costs attributed to these reached, on average, $35 million per year and more than $500 million lost from 2001 to 2017 (Table 4).

**Table 4:** Years of life lost (YLL) due to female homicide and femicide in Ecuador, 2001 and 2017

Year	Cases/year	YLL*	Economic losses (US$) **
2001	177	8840	13,903,903
2002	201	10,387	18,178,711
2003	170	8416	16,741,380
2004	211	10,717	21,319,347
2005	189	10,045	25,545,668
2006	219	11,501	29,601,214
2007	231	11,611	32,948,806
2008	200	10,256	36,835,534
2009	194	10,262	36,924,730
2010	189	9715	40,401,612
2011	226	11,222	53,612,987
2012	201	9956	49,850,610
2013	159	7751	40,789,337
2014	151	7457	41,455,658
2015	162	8076	39,547,989
2016	179	9242	47,448,887
2017	177	9025	50,143,307
Total	**3236**	**164,479**	**595,249,680**

^*^Based on Life Expectancy according to GBD2010, ** Human Capital Method

## Discussion

Gender–based violence is a leading cause of injury and disability worldwide. When such violence culminates in the death of a girl or woman, this has disastrous long-term consequences on communities and societies. Gender–based violence has a huge social and economic cost in Ecuador, estimated at $35 million per year, and an average of 9675 years of life lost prematurely per year.

This is the first report conducted on the state of female homicides and femicides in Ecuador using national population level data. Previous studies have used a combination of local networks and media to produce estimates of femicide in Ecuador [17, 18]. Such estimates may not be representative of the national population and may be open to unreliable reporting. The use of national official mortality statistics is a strength of this paper.

Consistent with other studies, females under the age of 35 were most affected, whilst lower education conferred a greater disadvantage. Although an explanation on the exact mechanism through which lower education influences the likelihood of gender-based violence could not be offered here, other studies suggest that poor access to, and participation in, education promote gender inequalities and female disempowerment [23].

Similar to previous studies, the region with the highest cumulative incidence rate was the Amazon region [17]. In 2011, The National Survey on family relations and gender violence against women reported that six out of ten women experienced gender-based violence, with those of Indigenous and Afro-Ecuadorian descent being most afflicted. This is consistent with the evidence presented here, which shows that the highest prevalence of female homicides and femicides occurred amongst Afro-Ecuadorian women. In contrast to the National Survey report, that found the highest rates of psychological, sexual and physical violence in Morona Santiago, Tungurahua and Pastaza [24] Our study shows higher rates of female homicides in the provinces of Sucumbios, Esmeraldas, Santo Domingo de los Tsachilas, Bolivar, and El Oro throughout the period of 2001 to 2017. Although most murders generally occurred in urban areas, a closer analysis at the provincial level showed that a number of cantons of smaller populations and higher rurality (such as those in Pastaza and Esmeraldas) had very high rates of female homicides. This could in part be explained by recent reports of civil violence at the Ecuadorian borders; it is also plausible that social norms inherent within the rural context, may provide agency for such acts of violence [25–27].

In terms of policy, The Inter-American Court of Human Rights affirm that States are obliged, under International Law, to adopt effective public policy in order to prevent acts of violence towards women before they result in a femicide [28]. It was not until 2014 that such actions were brought into discussion in Ecuador [29]. Prior to 2014, there was no criminal procedure to hold perpetrators accountable for attacks of violence and discrimination made against women and girls. One of the limitations of this study was that we were unable to differentiate between female homicides and femicides prior to 2014, when the legislation was put in place, and therefore the information collected from 2001 to 2013 was based on the deaths of women due to female homicides, which included unreported femicides. Another limitation was that some of the social, economic and demographic information reported here were based on reporting from close family members of the deceased and the professionals involved; this may have resulted in misclassifications or inaccuracies in reporting. A further limitation was that we lacked information on the perpetrator of the crime, which meant that we could not confirm the rates of intimate partner homicide [29]. According to the Ecuadorian State established a Gender Atlas, the most common perpetrators of femicides included people unknown to the victim (30%), cohabitant (30%), former partner (16%), family member (8%), boyfriend (7%), ex-boyfriend (2%) among others (28). Further studies using individual data should seek to address this data gap and should further seek to measure the underlying drivers of the provincial disparities.

Overall these findings demonstrate both the scale and impact of female homicides and femicides in Ecuador. Although not analysed here, other studies elsewhere have reported implementation problems following changes in legislation. Domestic violence laws may not be accompanied by the required budget allocations, for example, or certain authorities involved in law enforcement may be resistant [30]. A national strategy to tackle femicide, through community initiatives that address the risk factors of violence (such as the acceptability of violence) alongside better enforcement of laws is recommended. For example, group educational training that target inequitable gender norms, and address underlying expectations around female and male behaviour, have been shown to be effective within targeted populations in LMICs. Evidence also shows that community mobilization programmes—complex interventions involving many community stakeholders—can also promote changes in norms, discourse and practice through communication channels such as radio and television.

Femicide is a global human rights violation that until now has received very little attention and research in Ecuador. Policy makers should consider the devastating impact of femicides on the national economy, and fund further investment into the enforcement of new legislation, as well as national and local programmes that address gender inequality; thereby protecting the livelihood and well-being of their women and girls.

## Supplementary Information

**Additional file1: **ICD- CODES and categorisation used for mortality estimates.

## Data Availability

The entire data is here https://aplicaciones3.ecuadorencifras.gob.ec/sbi-war/ The datasets used and/or analysed during the current study available from the corresponding author on reasonable request.
