# Supplementary material for: Female homicides and femicides in Ecuador: a nationwide ecological analysis from 2001 to 2017
Source: BMC Womens Health. 2022 Jun 27;22:260. doi: 10.1186/s12905-022-01839-2 (PMC9238169; doi:10.1186/s12905-022-01839-2)
Supplement: Supplementary file 1 — Additional file1: ICD- CODES and categorisation used for mortality estimates. [file 12905_2022_1839_MOESM1_ESM.docx]

**Appendix: ICD- CODES and categorisation used for mortality estimates**

| **ICD- CODE** | **Description** | **Nine categories included and corresponding codes** |
| --- | --- | --- |
| X85 | Assault by drugs, medicaments and biological substances | Chemicals |
| X86 | Assault by corrosive substance |  |
| X87 | Assault by pesticides |  |
| X88 | Assault by gases and vapours |  |
| X89 | Assault by other specified chemicals and noxious substances |  |
| X90 | Assault by unspecified chemical or noxious substance |  |
| X91 | Assault by hanging, strangulation and suffocation | Strangulation |
| X92 | Assault by drowning and submersion | Drowning |
| X93 | Assault by handgun discharge | Firearms |
| X94 | Assault by rifle, shotgun and larger firearm discharge |  |
| X95 | Assault by other and unspecified firearm discharge |  |
| X96 | X96 Assault by explosive material | Fire-related mechanisms |
| X97 | Assault by smoke, fire and flames |  |
| X98 | Assault by steam, hot vapours and hot objects |  |
| X99 | Assault by sharp object | Sharp objects |
| Y00 | Assault by blunt object | Blunt objects |
| Y01 | Assault by pushing from high place | Included in ‘Others’ |
| Y02 | Assault by pushing or placing victim before moving object |  |
| Y03 | Assault by crashing of motor vehicle |  |
| Y04 | Assault by bodily force |  |
| Y05 | Sexual assault by bodily force | Sexual aggressions |
| Y06 | Neglect and abandonment | Included in ‘Others’ |
| Y07 | Other maltreatment |  |
| Y08 | Assault by other specified means |  |
| Y09 | Assault by unspecified means |  |
